# Metabolism drives distribution and abundance in extremophile fish

**DOI:** 10.1371/journal.pone.0187597

**Published:** 2017-11-27

**Authors:** Richard S. A. White, Peter A. McHugh, Chris N. Glover, Angus R. McIntosh

**Affiliations:** 1 School of Biological Sciences, University of Canterbury, Christchurch, New Zealand; 2 Department of Watershed Sciences, Utah State University and Eco Logical Research Inc., Logan, Utah, United States of America; 3 Athabasca River Basin Research Institute and Faculty of Science, Athabasca, Alberta, Canada; 4 School of Biological Sciences, University of Alberta, Edmonton, Alberta, Canada; University of Hyogo, JAPAN

## Abstract

Differences in population density between species of varying size are frequently attributed to metabolic rates which are assumed to scale with body size with a slope of 0.75. This assumption is often criticised on the grounds that 0.75 scaling of metabolic rate with body size is not universal and can vary significantly depending on species and life-history. However, few studies have investigated how interspecific variation in metabolic scaling relationships affects population density in different sized species. Here we predict inter-specific differences in metabolism from niche requirements, thereby allowing metabolic predictions of species distribution and abundance at fine spatial scales. Due to the differences in energetic efficiency required along harsh-benign gradients, an extremophile fish (brown mudfish, *Neochanna apoda*) living in harsh environments had slower metabolism, and thus higher population densities, compared to a fish species (banded kōkopu, *Galaxias fasciatus*) in physiologically more benign habitats. Interspecific differences in the intercepts for the relationship between body and density disappeared when species mass-specific metabolic rates, rather than body sizes, were used to predict density, implying population energy use was equivalent between mudfish and kōkopu. Nevertheless, despite significant interspecific differences in the slope of the metabolic scaling relationships, mudfish and kōkopu had a common slope for the relationship between body size and population density. These results support underlying logic of energetic equivalence between different size species implicit in metabolic theory. However, the precise slope of metabolic scaling relationships, which is the subject of much debate, may not be a reliable indicator of population density as expected under metabolic theory.

## Introduction

Understanding the drivers of species distribution and abundance is an important goal of predictive ecology [1]. Distribution and abundance is affected by both intrinsic species traits and environmental conditions [2], both of which are variable and species specific, making it difficult to apply general principles to predict abundance [3]. Because metabolic rate is a measurable trait all species and determines per capita consumption rates in populations and therefore can have effects on carrying capacity, metabolic rates are being increasingly used as general predictors of species abundance [4,5,6]. Metabolic rate is often shown to scale with body size according to a power law with an exponent of 0.75 a slope which is assumed to drive the negative power law scaling of population size with body size, which has a slope of -0.75 [4,5,7]. Because population size is limited by energy availability divided by per-capita consumption rates, as body size increases, per-capita consumption rate rise and energy availability must be divided amongst fewer individuals, thus resulting in negative scaling of abundance with body size.

While this metabolic concept has support at macroecological scales [4,8], many studies show significant departures in size-density relationship exponents from that expected from the scaling of metabolism with body size [9,10]. In particular, as body size range declines to that which is more likely at local scales, the residual variation in density that is unexplained by body size increases relative to that which is explained by body size [11], and the slopes of the size-density relationship fluctuate greatly [12]. Meanwhile variation in habitat productivity and food availability can also generate departures in size-density relationships from that expected by metabolic theory [6,13]. For animals that cannot manipulate their prey, for example, increases in gape size with body mass can increase food availability to larger individuals, which may alter size-density relationships within populations [14]. Finally, there is evidence that the exponent of the relationship between metabolic rate and body size varies widely depending on species and life-history [15,16,17,18]. Consequently, the applicability of body size as a universal predictor of density may be limited at small scales, where location and species-specific knowledge may be more insightful.

One way to improve local predictions from metabolic theory may be to consider how functional traits, such as metabolism, vary with environmental gradients to affect ecological function at finer spatial scales [2]. While the precise physiological mechanisms controlling the scaling of metabolic rate with body size are heavily debated [19,20], it is well known that metabolic rates vary independently of mass and that the scaling parameters can vary significantly from that expected from metabolic theory [17]. Higher metabolic rates are often associated with greater competitive, and aerobic capacity, dominance, aggression and growth rate [21,22,23,24], potentially leading to higher fitness [25,26]. However, such relationships are heavily context dependent [27], often due to limitations environments place on resource use and oxygen consumption [28,29]. For example, metabolic rates often scale negatively with environmental stresses including aridity, food scarcity and hypoxia [30,31,32,33,34]. Meanwhile systematic changes in exponents for the scaling of metabolism with body size have also been observed along environmental gradients associated with cold temperatures, and low food and oxygen availability [18,35]. Such systematic variation in metabolic traits may give rise to predictable changes in population density along environmental gradients [36] and improve predictions from metabolic theory at smaller spatial scales.

We examined whether interspecific differences in the scaling of metabolic rates with body size along an environmental gradient could account for variation in size-density relationships between species of freshwater fish. The brown mudfish (*Neochanna apoda*) and banded kōkopu (*Galaxias fasciatus*) are fish that are allopatrically distributed along an environmental stress gradient involving extreme drought, hypoxia and acidity in forest ponds [37]. Brown mudfish are restricted to hypoxic, acidic, drought-prone pools because of their vulnerability to predation by kōkopu in permanent pools [37]. Despite the harsh environment brown mudfish inhabit, they can reach remarkably high population densities, averaging more than 30 fish m^-3^ [38,39]. We predicted such high brown mudfish population densities resulted from low individual metabolic rates, which may have evolved in response to the hypoxic environment they inhabit, in contrast to the kōkopu which are usually found in much lower densities [37]. In particular, we tested the hypothesis that inter-specific differences in the intercept and slope of the relationship between mass and standard metabolic rate could explain inter-specific differences in body size-density relationships between mudfish and kōkopu.

## Materials and methods

### Study system

All procedures were approved by the University of Canterbury animal ethics committee (permit number 2010/23R). Fish abundance was determined in 39 mudfish and 25 kōkopu pools in the Saltwater Forest, Westland National Park, South Island, New Zealand during the austral summer of 2011–2012. Saltwater Forest is a low altitude (20–100 m ASL) 9000 ha temperate peat-swamp-rainforest with high annual rainfall (3742 mm) [40]. Poor soil drainage allows many small shallow pools to form on the forest floor, which may be permanently or intermittently flooded. Most pools are excavated by tree fall events, which uproot large amounts of soil, and generally do not exceed 0.3 m depth and 2.5 m^3^ in volume. Mudfish and kōkopu are allopatrically distributed within Saltwater Forest, with mudfish restricted to temporary, hypoxic (mean dissolved oxygen: 1.34 mg O_2_ L^-1^), or acidic pools (mean pH: 4.30), and kōkopu restricted to permanent pools with higher oxygen content (mean: 2.31 mg O_2_ L^-1^) and pH (mean: 4.74) [37]. Although these chemical conditions are significantly different according to past analyses, the probability of pool drying is the main physical stressor driving the allopatric distribution of mudfish and kōkopu [37]. Kōkopu are only found in permanent pools or flowing rivers, whereas mudfish may be found in pools that dry over twenty times a year [37,38,41].

### Population size estimation

Kōkopu and mudfish populations were located within four randomly positioned 100 × 20 m transects, which were stratified by altitude. Pool volumes were calculated by multiplying pool surface area (±0.01 m) by average pool depth over time (±0.01 m). Pool surface area was estimated using the formulas for an ellipse, square, circle or semi-circle depending on pool shape or some combination thereof for irregularly shaped pools. Surface areas and depths were measured for all pools on the same day to avoid bias caused by temporal variation in pool volume. Although kōkopu are also found in streams, brown mudfish are absent from streams in our study area, potentially due to poor swimming ability [37]. Thus we restricted our population sampling to lentic pools to avoid introducing potentially spurious lotic-lentic effects on population sizes. However, this made it difficult to locate a balanced number of populations for each species (i.e. 39 and 25 mudfish and kōkopu populations, respectively). Mudfish and kōkopu mass averaged 6.2 and 16.4 g and ranged between 0.2–20.6 and 0.5–68 g, respectively in the field.

Four mudfish and four kōkopu pools were randomly selected for continuous temperature measurement during the sampling period, using one WT-HR stage/temperature logger (TruTrack, Christchurch, New Zealand) per pool. Temperature was logged in at least one pool in each transect, and water temperature was recorded hourly in each of these pools from 29 November 2011 to 23 March 2012. Fish populations within pools were sampled using un-baited 3.12 mm (1/8”) mesh Gee minnow traps (420 mm L × 220 mm W) set for 12 h overnight at a constant density of 1 trap per 2 m^2^ of the pool surface area. Upon capture, all fish were anaesthetised with a 0.5 × 10^−5^ g L^-1^ concentration of 2-phenoxyethanol, weighed using a Scout Pro scale (±0.1 g) (Ohaus^®^, Pine Brook, North America), and their total length measured (±1 mm). All fish sampling and handling protocols were approved as part of obtaining the field permit granted by the Department of Conservation, New Zealand.

Total population biomass within each pool was estimated by summation of the weights of all individual fish caught in a pool on a single trapping night. Weights for individual fish were estimated using original length-wet mass regressions. Population biomass was divided by pool volume to estimate population biomass density (±0.1 g m^3^). To investigate the assumption that our population biomass density estimates were unbiased towards either species, we conducted a mark-recapture survey of a subset of 33 mudfish pools and 16 kōkopu pools. Pools were sampled using the same protocol as described above, but all fish caught on the first sample were uniquely marked using a combination of visual implant elastomer tags (Northwest Marine Technology, Inc, Shaw Island, North America) for fish <80 mm and passive inductive transponder (PIT) tags (Oregon RFID, Portland, USA) for fish >80 mm. Despite higher rates of tag loss being recorded in PIT tagged mudfish in past research [39], no evidence of tag loss was recorded in the present study and therefore no biases were introduced by using multiple tag types. Sampling was repeated following a three to five day interval and the number of marked and unmarked fish was counted. For each site, we compared the total density of fish caught on sample one (sample one density) with the total density of unique fish from samples one and two (total population density). A homogeneity of slopes test showed that there was no significant difference in the slope of the relationship between total population densities and the sample one densities for mudfish and kōkopu (*R*^2^ = 0.97, *F*_1,45_ = 0.0004, *P* = 0.98); both slopes were approximately 0.95 ± 0.025 g m^3^ (S1 Fig). Thus the densities estimated from a single trapping event equated to approximately 95% of the total population densities for both species and was unbiased.

### Metabolic rates

Standard metabolic rates (SMR) and maximum metabolic rates (MMR) were measured on 26 and 29 brown mudfish and banded kōkopu, respective, by measuring the rate of oxygen uptake using closed box respirometry before and after exhaustive exercise in laboratories at the University of Canterbury. Mudfish and kōkopu mass averaged 5.8 and 7.5 g and ranged between 0.4–15.9 and 0.5–24.8 g, respectively. Both fish species are benthic and remained rested on the bottom of the respirometers during measurement. Brown mudfish and banded kōkopu were caught using Gee minnow traps (GMT) placed overnight in pools and streams located in the West Coast region, near Hokitika New Zealand. Fish were held in static 20-L plastic containers containing aerated freshwater, maintained at constant temperature (14°C) and light conditions (12 h: 12 h). Fifty percent water changes were made daily and fish were fed *ad libitum* on commercial bloodworms (Aqua One^®^, Sydney, Australia), until three days before experimentation. Individual fish were acclimated to their respirometers (0.10 L, 0.25 L, 0.50 L or 1 L glass Schott bottles [Schott^®^, Elmsford, North America], depending on fish mass) for 12 h overnight with continuous water flow prior to measurement. The ratio between fish and respirometer volume averaged approximately 0.02 g L^-1^. The respirometers were immersed in a water bath maintained at 14°C at all times, and were sealed using rubber bungs after acclimation, and the water bath was covered by black mesh to minimise visual disturbance.

Previous respirometry studies on Galaxiidae species have used a closed respirometry procedure [42,43,44,45] based on that of Sloman et al. (2006) [46]. This system allows the use of relatively small respirometers (min = 0.065 L) which has proven the most practical for measuring metabolism in small (often 0.4 g), slowly respiring Galaxiidae species, whose oxygen consumption rates can be otherwise difficult to detect relative to measurement error, in larger respirometers [47,48]. It has been reported that closed respirometry systems may introduce metabolic artefacts associated with hypoxia and hypercapnia [48]. However, a test of the effect of carbon dioxide accumulation using closed respirometry in another galaxiid fish, showed that elevated carbon dioxide had no impact on oxygen consumption [45]. Consequently, this closed system has provided reliable results over multiple independent studies on Galaxiidae species [42,43,44], including studies done in semi-closed respirometers [49]. To ensure our results were comparable to these studies on Galaxiidae, we used identical closed respirometry methods found therein based on the work of Sloman and colleagues (2006).

Standard metabolic rate was measured by comparing the change in oxygen concentration between water samples (0.7 ml) taken at 20 minute intervals from each respirometer. The small volume of water removed was replaced to avoid unwanted bubble formation in respirometers. Samples were withdrawn with a syringe and injected into an MC100 microcell (Strathkelvin Instruments^®^, Glasgow, Scotland) containing an SI 130 oxygen electrode (Strathkelvin Instruments^®^, Glasgow, Scotland). The oxygen electrode was connected to an oxygen meter (SK Model 781, Strathkelvin Instruments^®^, Glasgow, Scotland), with output recorded on a computer via a Powerlab 4/SP (ADInstruments^®^, Richmond-Windsor, Australia). The electrode was calibrated daily before measurements using fully aerated water and a saturated sodium sulphite solution. Water from the water bath was pumped through the microcell water jacket so that water samples were maintained at 14°C during measurement. Respirometers were sealed during the entire procedure and measurements were only made above *P*_*O2*_ = 100 mmHg (~6.6 mg O_2_ L^-1^) to avoid the effects of hypoxia on oxygen consumption. The rate of oxygen consumption was calculated as:

$$MO_{2}=\frac{\Delta O_{2i}\cdot \alpha O_{2}\cdot \mu O_{2}\cdot V_{i}}{\Delta T}$$

where Δ*O*_2*i*_ is the change in oxygen partial pressure (torr) in fish *i*’s respirometer between samples, *αO*_*2*_ is a constant reflecting the solubility of O_2_ in freshwater at 14°C (2.0518 μmol L^-1^ torr^-1^), *μO*_*2*_ is the molecular mass of oxygen (31.99 μ), *V*_*i*_ is the volume (L) of fish *i*’s respirometer and Δ*T* is the time interval between samples. Three SMR samples were taken over an hour (three 20 min samples) for each fish, the mean of which was use as the final SMR estimate for each fish. Fishless controls were run concurrently for all experiments, however, no microbial oxygen consumption was detected.

A subset of 24 mudfish and 19 kōkopu were then measured for their MMR following determination of their SMR. MMR was determined after individuals were exhaustively exercised using a forced swimming protocol similar to that of MacKenzie and colleagues [50]. Approximately one hour after SMR measurement, individual fish were transferred from their respirometers to a separate water bath maintained also at 14°C and then manually chased for 15–20 minutes until exhaustion. Fish were considered to be exhausted when they were no longer capable of escaping by burst swimming and could only make weak body movements. Immediately after exhaustion, fish were returned to their respirometers and MMR was measured using the same protocol described for SMR. However, for MMR, we used a 15-minute sample interval, and metabolism was repeatedly measured until SMR levels were reached. We used the oxygen consumption measured during the first period following exercise as the measure of MMR, which was the highest value measured for each fish. Fish were then weighed in grams (± 0.01) and returned to their aquaria. Aerobic scope (AS) was then calculated as MMR_*i*_/SMR_*i*_ which represents the factorial increase of MMR over SMR for fish *i*.

### Metabolic scaling

SMR and MMR values were calculated as mg O_2_ h^-1^, thus all values were absolute rather than mass-specific unless otherwise stated. SMR, MMR and AS values were all log_10_-transformed for each fish (to ensure linearity for linear regression analyses) and were regressed against the log_10_ of the fish mass (g). A homogeneity of slopes test was first used to test for species-specific differences in slopes using an interaction between species identity and the log_10_ of fish mass for all metabolic metrics. If interaction terms were not significant, we ran an ANCOVA with interaction terms removed.

### Effects of metabolic rates on population densities

The SMR scaling relationships determined above for mudfish and kōkopu were used to estimate the metabolic rate of each fish caught in each population based on their mass and species identity and was treated as mass-specific metabolic rate (i.e. the total metabolic rate of each fish in each population was divided by its mass). A one-way ANOVA showed that the average daily maximum temperature in mudfish pools (13.9 ± 0.4°C) was not significantly different to that in kōkopu pools (13.5 ± 0.5°C, *F*_1,6_ = 0.46, *P* = 0.52), and was close to the temperature used to derive the SMR scaling relationship (14°C). Thus, temperature corrections were not applied to our population SMR estimates. The log_10_ of the average SMR of individuals in each population (log[μSMR]) was then calculated from these data. The log_10_ of the average mass of individuals in each population (log[μMASS]) was also calculated. Thus μMASS and μSMR population-level measures of fish mass and SMR calculated for the average fish in each population, respectively. Finally we calculated the log_10_ population biomass density for each pool sampled.

We were interested in the relative variance in population biomass density explained by log(μSMR) compared to the variance explained by the combination of species identity, log(μMASS) and their interaction. If interspecific variation in metabolism was responsible for interspecific variation in population biomass density, then log(μSMR) should explain the same variation in density as the combination of log(μMASS), species identity and their interaction. In this case, log(μSMR) would be the only variable necessary, and log(μMASS) and species identity would be removed in the process of model simplification. Thus we ran two model simplification analyses starting with two alternative full ANOVA models that depicted two cases. In the body mass model, where log(μSMR) was excluded, the starting full model included species identity and log(μMASS) direct effects and their interaction as predictors of log population biomass density. In the metabolism model we included these effects in addition to the main effect of log(μSMR), and its interaction with species identity. Predictive terms were removed from each model starting with non-significant interaction terms, followed by the least significant main effects based on type II sums of squares ANOVA which accounts for potential co-linearity between terms [51]. All models satisfied the assumptions of a parametric ANOVA once both axes were log_10_ transformed. Terms were left out if their removal resulted in a lower AIC_c_ (Akaike’s information criterion corrected for small sample size). Finally, we compared the strength of support for the two simplified models using *R*^2^, AIC_c_, and Akaike weights (*w*). All analyses were performed in R 3.0.1 (R Foundation for Statistical Computing, Vienna, Austria).

## Results

### Standard metabolic rate

**Fig 1:**
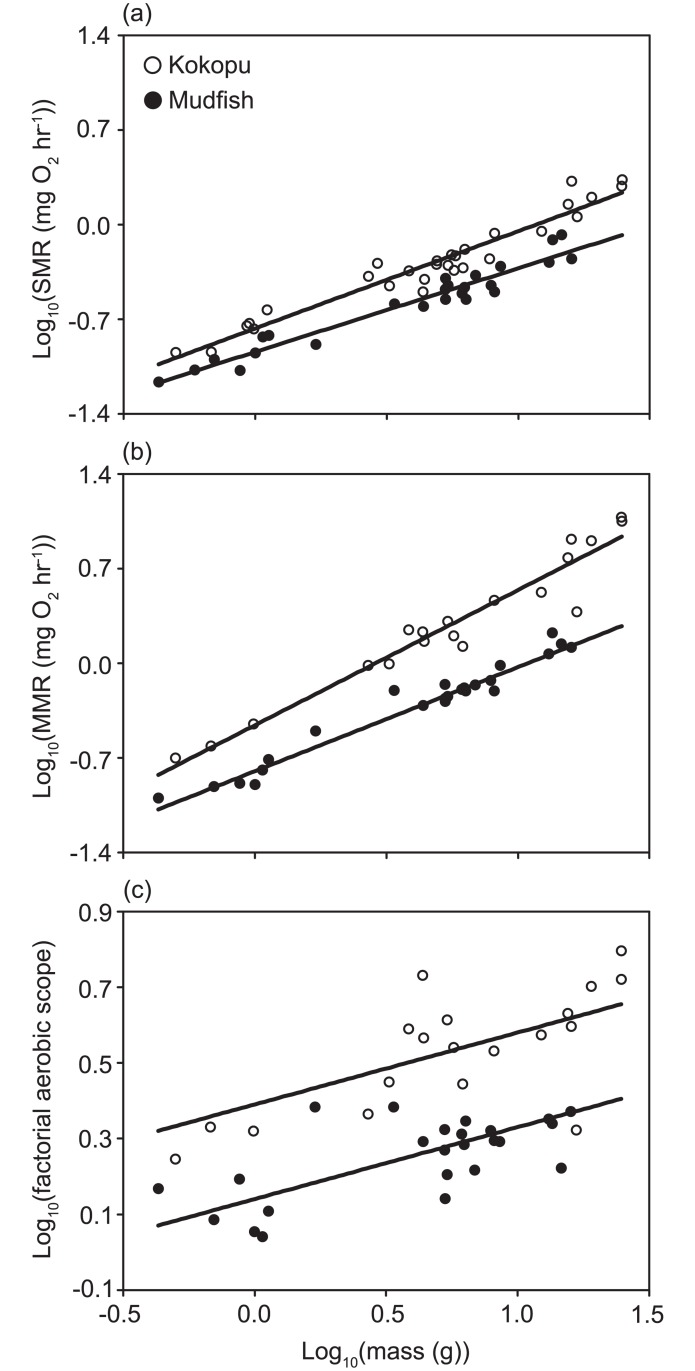
The effect of fish mass, on (a) standard metabolic rates (SMR), (b) maximum metabolic rates (MMR) and (c) factorial aerobic scope (i.e. MMR_i_/SMR_i_ for fish i), for mudfish (closed circles) and kōkopu (open circles). All data points are for individual fish. The X axes are identical for all plots and y axes are identical for plot (a) and (b) and all are log_10_-transformed.

Mudfish and kōkopu SMR averaged 0.32 (± 0.04) and 0.70 (± 0.04) mg O_2_ h^-1^ and ranged between 0.1–0.8 and 0.1–2.1 mg O_2_ h^-1^, respectively and increased with mass for both species. The slope of the relationship between SMR and mass was significantly greater for banded kōkopu than for mudfish (Homogeneity of slopes test: *F*_1,50_ = 4.53, *P* = 0.038) (Fig 1a). Thus for mudfish, and banded kōkopu, the relationships between SMR and mass were best described by the equations: *SMR*_*mudfish*_ *=* 0.11*Mass*^0.62(±0.03)^ and *SMR*_*kōkopu*_ = 0.17*Mass*^0.72(±0.03)^, where SMR is in mg O_2_ h^-1^. The average mass of all fish measured for SMR was 6.7 g. At this weight, these equations predict kōkopu will consume 1.9 times more oxygen (0.67 mg O_2_ h^-1^) than mudfish (0.36 mg O_2_ h^-1^) at rest.

### Maximum metabolic rate

Mudfish and kōkopu SMR averaged 0.6 (± 0.03) and 3.5 (± 0.85) mg O_2_ h^-1^ and ranged between 0.1–1.7 and 0.2–11.9 mg O_2_ h^-1^, respectively and increased with mass for both species. The slope of the relationship between MMR and mass was significantly greater for kōkopu than mudfish (Homogeneity of slopes test: *F*_1,39_ = 10.93, *P* < 0.01) (Fig 1b). Thus for mudfish and kōkopu, the relationships between MMR and were best described by the equations: *MMR*_*mudfish*_ = 0.16*Mass*^0.77(±0.05)^ and *MMR*_*kōkopu*_ = 0.35*Mass*^1.0(±0.05)^, where MMR is in mg O_2_ h^-1^. Thus at 6.7 g, these equations predict kōkopu will maximally consume 3.4 times more oxygen (2.35 mg O_2_ h^-1^) than mudfish (0.69 mg O_2_ h^-1^).

### Aerobic scope

Mudfish and kōkopu factorial aerobic scope averaged 1.8 (± 0.03) and 3.6 (± 0.29) times SMR and ranged between 1.1–2.4 and 1.8–6.3 times SMR, respectively and increased with mass for both species. There were no differences in the slopes of the relationship between AS and mass for BM or BK (Homogeneity of slopes test: *F*_1,39_ = 1.50, *P* = 0.23) and AS was significantly lower for BM than BK for all sizes (ANCOVA species effect: *F*_1,40_ = 69.43, *P* < 0.001) (Fig 1c). Thus for BM and BK, the relationship between AS and mass was best described by the equations: *AS*_*mudfish*_ = 1.38*Mass*^0.19^ and *AS*_*kōkopu*_ = 2.45*Mass*^0.19^, where *y* is AS (i.e. MMR/SMR). Thus at 6.7 g, these equations predict that kōkopu can more than triple its SMR (3.5x SMR) if needed, whereas mudfish can only double theirs (2.0 × SMR) if needed, which is a 1.75-fold difference.

### Effects of metabolism on population density

Mudfish and kōkopu population sizes averaged 8.8 and 6.4 and ranged between 1–42 and 1–20, respectively. Mudfish had a lower mass-specific SMR than kōkopu and thus, according to MTE, they should have a higher mass-specific population density, which would require a species identity term to explain differences in population density (i.e. density = log(μMASS) × species identity). If mass-specific SMR differences were responsible for the mass-specific density difference, then a model that additionally included species mass-specific log(μSMR) would explain the same variation but species identity and mass effects would become redundant.

**Fig 2:**
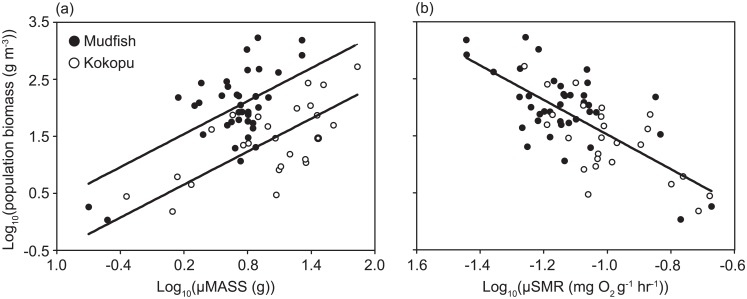
The effects of (a) the average mass of individuals in a population (log[μMASS]) in g and (b) the average standard metabolic rate (SMR) of individual fish in a population (log[μSMR]) in mg O_2_ g^-1^ h^-1^, on the density of biomass in mudfish (closed circles) and kōkopu (open circles) populations. All data points are for individual populations, and all axes are log_10_ transformed.

In the body mass model, where the effect of log(μSMR) was excluded, the minimal adequate model that explained variation in mudfish and kōkopu population density included both the direct effects of log(μMASS) and species identity (Table 1). Population biomass density was significantly positively related to Log(μMASS) (mass effect: *F*_1,61_ = 46.11, *P* < 0.001), with a slope of 0.96, which did not differ significantly between species (species × mass interaction: *F*_1,60_ = 1.44, *P* = 0.24) (Fig 2a). However, the intercept for this relationship was significantly higher for mudfish populations (species effect: *F*_1,61_ = 42.24, *P* < 0.001) (Fig 2a). Excluding species identity resulted in a 36 percent reduction in the model *R*^2^ from 0.52 to 0.19, and a large 30.56 unit increase in AIC_c_. Thus there were large differences in population biomass densities between mudfish and kōkopu that could not be explained by fish mass (Fig 2a).

**Table 1:** Model selection results for the body mass model (i.e. excluding species SMR differences) and the metabolic model (i.e. incorporating species SMR differences) evaluating the metabolic controls on mudfish and kōkopu population biomass density. *R*^2^ is the coefficient of determination, AIC_c_ is Akaike's information criterion corrected for small sample size and *w* is the Akaike weight for the models that were simplified (i.e. all non significant terms removed) from their corresponding full model. Log(μMASS) and log(μSMR) are the average mass and standard metabolic rates of individual fish in a population in g and mg O_2_ g^-1^ h^-1^, respectively. Species identity is a two-level factor (banded kōkopu or brown mudfish). A ‘×’ denotes an interaction between a factor and all subsequent variables in brackets.

Model	Full model	Simplified model	*R*^2^	AIC_c_	*w*
1	Species identity × (log[μMASS])	log(μMASS) + species	0.52	98.5	0.4
2	Species identity × (log[μMASS] + log[μSMR])	log(μSMR)	0.51	97.6	0.6

In the metabolic model, that included the effect of differences in mudfish and kōkopu log(μSMR), the minimal adequate model that explained variation in mudfish and kōkopu population density included only the single effect of log(μSMR) (Table 1). Log(μSMR) was significantly negatively related to population biomass density (mass effect: *F*_1,62_ = 65.17, *P* < 0.001), with a slope of –3.07, which was identical for both species (mass × species interaction: *F*_1,60_ = 0.069, *P* = 0.79) and had an *R*^2^ of 0.51 (Fig 2b). In contrast to the body mass model, there were no significant differences in population biomass density between species when density was regressed with log(μSMR) (Fig 2a and 2b) (species effect: *F*_1,61_ = 1.79, *P* = 0.19). Thus mudfish and kōkopu population densities were similar when comparing populations with similar average individual metabolic rates, but not when comparing populations with similar sized fish (Fig 2a and 2b). In fact, removal of log(μMASS) and species identity terms reduced the total model *R*^2^ by only 0.04 units and the model AIC_c_ by 5.63 units. Consequently, the variance explained by log(μSMR) in the metabolic model was virtually identical to that explained by the combination of species identity and log(μMASS) in the body mass model (Table 1). Thus, the model using only log(μSMR) was the most parsimonious, and explained over 50 percent of the variation in population biomass density in a single variable (Fig 2b). Consequently, the differences in mass specific population density between mudfish and kōkopu were explained by species mass-specific SMR differences.

## Discussion

The assumption that metabolic rate increases with body size with a slope of 0.75 is often made when explaining variation in population density between different sized species [4,5,7,8]. Many authors have criticised this assumption, highlighting that quarter power scaling of metabolic rate with body size is not universal and there is significant interspecific variation in metabolic scaling parameters between taxa [9,10,17]. However few studies have investigated whether interspecific variation in metabolic scaling parameters yield predictable differences in population density between species. Here we found significant differences in metabolic rates between brown mudfish and banded kōkopu, which helped explain interspecific variation in population density, but not in a way that was consistent with 0.75 scaling. In particular, brown mudfish had a significantly lower standard metabolic rate compared to banded kōkopu, which appeared to have enabled them to reach higher population densities. Nevertheless, despite significant differences in the slope of mudfish and kōkopu metabolic scaling relationships, the relationships between body size and population density were equivalent between the two species. These findings suggest that interspecific differences in metabolism can be a useful indicator of differences in population density between species, thus supporting the underlying logic of metabolic theory that species metabolism is an important driver of ecological function. However, our result ssuggest the precise slope of metabolic scaling relationships, which is the subject of much debate [14], may not be a reliable indicator of population density as expected under metabolic theory.

The fact that the slopes of the relationship between body size and population density were equivalent between mudfish and kōkopu, despite significant differences in metabolic scaling exponents, suggests that allometric scaling factors other than metabolism were involved in determining population density. In our case, population density increased with body size more rapidly than expected from the mudfish and kōkopu metabolic scaling exponents. This may occur if food availability also increased with body size due to the reduction in gape size limitation that occurs with mass [52], which can cause a greater than expected slope for the relationship between body size and population density [9,14]. We observed prey in our pools ranging from small amphipods (<5 mm long), through to moderately sized odonates and large terrestrially-derived *Prionoplus reticularis* beetle adults (New Zealand’s largest terrestrial beetle) [53]. Thus, although food availability was unlikely to have varied systematically with species across our sites, there was a large range of prey sizes which would be made available during ontogeny, within sites. Greater potential food availability for larger individuals may explain why the slope of the relationship between body size and population density was steeper than expected from each species metabolic scaling parameters (i.e. why there were more large individuals supported than expected from their metabolic rates). This illustrates one of the many factors other than metabolism, that potentially contribute to variations in population density, and which would likely confound the allometric scaling predictions of metabolic theory.

Regardless of the lack of correspondence between the metabolic and population density scaling exponents, it is interesting that the overall interspecific differences in metabolic rates and population density were directly proportional. Mudfish mass-specific population density was higher compared to that of kōkopu for the range of fish sizes surveyed because mass did not account for inter-specific variation in metabolic rate. Differences in species intercepts for the relationship between mass and density disappeared when species mass-specific metabolic rate was used to predict density. Thus, mudfish and kōkopu population sizes and total energy use were equivalent once their metabolic differences were accounted for. It is possible, however, that higher food availability in mudfish populations could also explain the higher mudfish population densities, as has been shown for mammals [6]. Populations in this study were chosen to minimise the physical differences between mudfish and kōkopu habitat (i.e. only lentic pools were chosen). Although mudfish pools were significantly more prone to drying and were more acidic and hypoxic [37], these stressors are likely to cause higher mudfish mortality resulting in lower population density compared to banded kōkopu, which was not the case. However, it is difficult to exclude the possibility of differences in food availability between species given that such data was not measured. Measuring productivity in these populations is challenging given that allochthonous detritus and invertebrate subsidies likely represents the greatest source of energy therein [54]. Recent invertebrate surveys conducted on fifteen of the mudfish pools used in this study indicated that total aquatic invertebrate community biomass averaged only 0.0019 g m^-3^ (± 0.0005 S.E.) (H. Warburton, pers. com.). Although these estimates were made using dry invertebrate body masses, it is likely that additional allochthonous invertebrate inputs would be required to sustain the total mudfish population biomasses reported here, which averaged 246 g m^-3^ (± 61 S.E.). Even so, low metabolic rates are likely also necessary to sustainably achieve such high biomass. Previous research using the same populations studied herein showed no significant differences in canopy cover between each species suggesting such allochthonous subsidies are likely to be equivalent between species [37]. Thus although we cannot rule out the possibility of other factors being responsible for the higher mudfish densities, it seems likely that low mudfish metabolic rate played at least some role.

In addition to the potential influence on population density, the interspecific difference in mudfish and kōkopu metabolic rates may play an important role in their driving their allopatric distribution associated with environmental harshness. Brown mudfish are generally only found in lentic pools that may dry up to 20 times a year and are hypoxic and acidic [37,38,54]. A low metabolic rate would enhance their survival in such conditions allowing them limited need for anaerobic respiration, which is costly and may not be sustainable under times of starvation during drought [43]. The ability of mudfish to extract oxygen under drought and hypoxia is likely enhanced by cutaneous respiration, which can comprise up to 43 percent of total respiration in closely related Canterbury mudfish (*Neochanna burrowsius*) by virtue of their scaleless integument, as comes standard with fish in the Galaxiidae family [43]. With nearly double the resting metabolic rate, kōkopu may be unable to survive such harsh conditions, despite also being scaleless, thus explaining their restriction to permanent pools and allopatry with mudfish [37]. At approximately 0.058 mg O_2_ g^-1^ h^-1^, brown mudfish routine metabolic rate was consistent with previous studies on brown mudfish SMR [44], and approximately equivalent to closely related Canterbury mudfish [51]. Mudfish SMR is 1.7–2.4 times lower than other members of the Galaxiidae family, including *Galaxias brevipinnis* (0.102 mg O_2_ g^-1^ h^-1^), *Galaxias vulgaris* (0.117 mg O_2_ g^-1^ h^-1^) and *Galaxias maculatus* (0.141 mg O_2_ g^-1^ h^-1^) which, like kōkopu, typically inhabit normoxic flowing streams and rivers and struggle to survive hypoxia and drought [42,55,56]. As our study suggests, such an environment is better suited to species with high aerobic scope which could enhance growth, reproductive potential and swimming ability.

Species similar to mudfish, such as African lungfish (*Protopterus sp*.), also adopt a low metabolic rate during droughts, however, unlike mudfish, lungfish undergo metabolic depression [57]. This involves suppressing metabolic rate [57], perhaps via mechanisms such as channel arrest during the drought [33,35], whilst burrowing and secretion of a moist cocoon. Although mudfish also burrow to maintain moisture during drought, they are incapable of sustained metabolic depression and instead adopt a consistently low metabolic rate during both emersion and immersion [44]. The process of metabolic depression in African lungfish involves substantial biochemical reorganisation and cocoon formation, taking days to accomplish and may last for up to 5 y [58]. Such a lengthy process may be too costly in mudfish habitats which can dry multiple times a week and may only last several days [38,41]. For mudfish, it may be more beneficial to pre-empt droughts by maintaining a constantly low metabolic rate. This hypothesis is supported by the fact that mudfish resume normal swimming activity almost immediately following weeks of emersion [44,55] indicating they are constantly primed for rapid, unpredictable changes between dry and wet conditions as is the hydrological nature of their pools. Such a strategy may have evolved in response to the relatively unpredictable weather patterns in New Zealand relative to larger continents where drying is more seasonal and predictable [59] thus allowing timing of metabolic depression such as is found with African lungfish.

## Supporting information

S1 Fig The relationship between the total population density (unique fish biomass from sample one + sample two) and the population density caught on the first sample. (DOC)

## References

[1] Guisan A, Thuiller W (2005) Predicting species distribution: Offering more than simple habitat models. Ecology Letters 8: 993–1009. 10.1111/j.1461-0248.2005.00792.x 34517687

[2] McGill BJ, Enquist BJ, Weiher E, Westoby M (2006) Rebuilding community ecology from functional traits. Trends in Ecology and Evolution 21: 178–185. doi: 10.1016/j.tree.2006.02.002 16701083 10.1016/j.tree.2006.02.002

[3] Lawton JH (1999) Are there general laws in ecology? Oikos 84: 177–192.

[4] Damuth J (1981) Population density and body size in mammals. Nature 290: 699–700.

[5] Enquist BJ, Brown JH, West GB (1998) Allometric scaling of plant energetics and population density. Nature 395: 163–165.

[6] Carbone C, Gittleman JL (2002) A common rule for the scaling of carnivore density. Science 295: 2273–2276. doi: 10.1126/science.1067994 11910114 10.1126/science.1067994

[7] Brown JH, Gillooly JF, Allen AP, Savage VM, West GB (2004) Toward a metabolic theory of ecology. Ecology 85: 1771–1789.

[8] Nee S, Read AF, Greenwood JJD, Harvey PH (1991) The relationship between abundance and body size in British birds. Nature 351: 312–313.

[9] Blackburn TM, Gaston KJ (1999) The relationship between animal abundance and body size: A review of the mechanisms. Advances in Ecological Research 28: 181–210.

[10] Loeuille N, Loreau M (2006) Evolution of body size in food webs: does the energetic equivalence rule hold? Ecology Letters 9: 171–178. doi: 10.1111/j.1461-0248.2005.00861.x 16958882 10.1111/j.1461-0248.2005.00861.x

[11] Isaac NJB, Storch D, Carbone C (2013) The paradox of energy equivalence. Global Ecology and Biogeography 22: 1–5.

[12] Isaac NJB, Storch D, Carbone C (2011) Taxonomic variation in size-density relationships challenges the notion of energy equivalence. Biology Letters 7: 615–618. doi: 10.1098/rsbl.2011.0128 21450722 10.1098/rsbl.2011.0128 PMC3130248

[13] Comor V, Thakur MP, Berg MP, de Bie S, Prins HHT, Van Langevelde F (2014) Productivity affects the density-body mass relationship of soil fauna communities. Soil Biology and Biochemistry 72: 308–311.

[14] Begon M, Firbank L, Wall L (1986) Is there a self-thinning rule for animal populations? Oikos 46: 122–124.

[15] Bokma F (2004) Evidence against universal metabolic allometry. Functional Ecology 18: 184–187.

[16] Glazier DS (2005) Beyond the '3/4-power law': Variation in the intra- and interspecific scaling of metabolic rate in animals. Biological Reviews of the Cambridge Philosophical Society 80: 611–662. doi: 10.1017/S1464793105006834 16221332 10.1017/S1464793105006834

[17] Glazier DS (2006) The 3/4-power law is not universal: Evolution of isometric, ontogenetic metabolic scaling in pelagic animals. BioScience 56: 325–332.

[18] Killen SS, Atkinson D, Glazier DS (2010) The intraspecific scaling of metabolic rate with body mass in fishes depends on lifestyle and temperature. Ecology Letters 13: 184–193. doi: 10.1111/j.1461-0248.2009.01415.x 20059525 10.1111/j.1461-0248.2009.01415.x

[19] Glazier DS (2010) A unifying explanation for diverse metabolic scaling in animals and plants. Biological Reviews 85: 111–138. doi: 10.1111/j.1469-185X.2009.00095.x 19895606 10.1111/j.1469-185X.2009.00095.x

[20] West GB, Brown JH, Enquist BJ (1997) A general model for the origin of allometric scaling laws in biology. Science 276: 122–126. 9082983 10.1126/science.276.5309.122

[21] DeLong JP (2008) The maximum power principle predicts the outcomes of two-species competition experiments. Oikos 117: 1329–1336.

[22] Stoffels RJ (2015) Physiological trade-offs along a fast-slow lifestyle continuum in fishes: What do they tell us about resistance and resilience to hypoxia? PLoS ONE 10. 10.1371/journal.pone.0130303 PMC4466508 26070078

[23] Réale D, Garant D, Humphries MM, Bergeron P, Careau V, Montiglio PO (2010) Personality and the emergence of the pace-of-life syndrome concept at the population level. Philosophical Transactions of the Royal Society B: Biological Sciences 365: 4051–4063. 10.1098/rstb.2010.0208 PMC2992747 21078657

[24] Biro PA, Stamps JA (2010) Do consistent individual differences in metabolic rate promote consistent individual differences in behavior? Trends in Ecology & Evolution 25: 653–659. 20832898 10.1016/j.tree.2010.08.003

[25] Brown JH, Marquet PA, Taper ML (1993) Evolution of body size: consequences of an energetic definition of fitness. American Naturalist 142: 573–584. doi: 10.1086/285558 19425961 10.1086/285558

[26] Lotka AJ (1922) Contribution to the Energetics of Evolution. Proceedings of the National Academy of Sciences of the United States of America 8: 147–151. 16576642 10.1073/pnas.8.6.147 PMC1085052

[27] Burton T, Killen SS, Armstrong JD, Metcalfe NB (2011) What causes intraspecific variation in resting metabolic rate and what are its ecological consequences? Proceedings of the Royal Society of London B Biological Sciences 278: 3465–3473. 10.1098/rspb.2011.1778 PMC3189380 21957133

[28] Hoffmann AA, Parsons PA (1989) Selection for increased desiccation resistance in *Drosophila melanogaster*: additive genetic control and correlated responses for other stresses. Genetics 122: 837–845. 2503423 10.1093/genetics/122.4.837 PMC1203758

[29] Hoffmann AA, Parsons PA (1989) An integrated approach to environmental stress tolerance and life-history variation: desiccation tolerance in Drosophila. Biological Journal of the Linnean Society 37: 117–136.

[30] Mueller P, Diamond J (2001) Metabolic rate and environmental productivity: Well-provisioned animals evolved to run and idle fast. Proceedings of the National Academy of Sciences of the United States of America 98: 12550–12554. doi: 10.1073/pnas.221456698 11606744 10.1073/pnas.221456698 PMC60091

[31] Lovegrove BG (2000) The zoogeography of mammalian basal metabolic rate. American Naturalist 156: 201–219. doi: 10.1086/303383 10856202 10.1086/303383

[32] Lovegrove BG (2003) The influence of climate on the basal metabolic rate of small mammals: A slow-fast metabolic continuum. Journal of Comparative Physiology B: Biochemical, Systemic, and Environmental Physiology 173: 87–112. doi: 10.1007/s00360-002-0309-5 12624648 10.1007/s00360-002-0309-5

[33] Hochachka PW (1986) Defense strategies against hypoxia and hypothermia. Science 231: 234–241. 2417316 10.1126/science.2417316

[34] Killen SS, Glazier DS, Rezende EL, Clark TD, Atkinson D, Willener AST, et al (2016) Ecological influences and morphological correlates of resting and maximal metabolic rates across teleost fish species. American Naturalist 187: 592–606. doi: 10.1086/685893 27104992 10.1086/685893

[35] Hochachka PW (1988) Channels and pumps-determinants of metabolic cold adaptation strategies. Comparative Biochemistry and Physiology Part B Biochemistry & Molecular Biology 90: 515–519.

[36] Lighton JRB, Brownell PH, Joos B, Turner RJ (2001) Low metabolic rate in scorpions: Implications for population biomass and cannibalism. Journal of Experimental Biology 204: 607–613. 11171311 10.1242/jeb.204.3.607

[37] White RSA, McHugh PA, Glover CN, McIntosh AR (2015) Multiple environmental stressors increase the realised niche breadth of a forest-dwelling fish. Ecography 38: 154–162.

[38] White RSA, McHugh PA, McIntosh AR (2016) Drought-survival is a threshold function of habitat size and population density in a fish metapopulation. Global Change Biology 22: 3341–3348. doi: 10.1111/gcb.13265 26929393 10.1111/gcb.13265

[39] White RSA, McHugh PA, Glover CN, McIntosh AR (2015) Trap-shyness subsidence is a threshold function of mark-recapture interval in brown mudfish *Neochanna apoda* populations. Journal of Fish Biology 87: 967–980. doi: 10.1111/jfb.12770 26376610 10.1111/jfb.12770

[40] Rogers HM (1999) Stand dynamics of *Dacrydium cupressinum* dominated forest on glacial terraces, south Westland, New Zealand. Forest Ecology and Management 117: 111–128.

[41] White RSA, Wintle BA, McHugh PA, Booker DJ, McIntosh AR (2017) The scaling of population persistence with carrying capacity does not asymptote in populations of a fish experiencing extreme climate variability. Proceedings of the Royal Society B: Biological Sciences 284. 10.1098/rspb.2017.0826 PMC5474084 28615503

[42] Urbina MA, Glover CN (2013) Relationship between fish size and metabolic rate in the oxyconforming inanga Galaxias maculatus reveals size-dependent strategies to withstand hypoxia. Physiological and Biochemical Zoology 86: 740–749. doi: 10.1086/673727 24241070 10.1086/673727

[43] Urbina MA, Meredith AS, Glover CN, Forster ME (2014) The importance of cutaneous gas exchange during aerial and aquatic respiration in galaxiids. Journal of Fish Biology 84: 759–773. doi: 10.1111/jfb.12303 24417441 10.1111/jfb.12303

[44] Urbina MA, Walsh PJ, Hill JV, Glover CN (2014) Physiological and biochemical strategies for withstanding emersion in two galaxiid fishes. Comparative Biochemistry and Physiology -Part A: Molecular and Integrative Physiology 176: 49–58. 10.1016/j.cbpa.2014.07.006 25026541

[45] Urbina MA, Glover CN, Forster ME (2012) A novel oxyconforming response in the freshwater fish Galaxias maculatus. Comparative Biochemistry and Physiology—A Molecular and Integrative Physiology 161: 301–306. 10.1016/j.cbpa.2011.11.011 22138470

[46] Sloman KA, Wood CM, Scott GR, Wood S, Kajimura M, Johannsson OE, et al (2006) Tribute to R. G. Boutilier: The effect of size on the physiological and behavioural responses of oscar, *Astronotus ocellatus*, to hypoxia. Journal of Experimental Biology 209: 1197–1205. doi: 10.1242/jeb.02090 16547292 10.1242/jeb.02090

[47] Svendsen MBS, Bushnell PG, Christensen EAF, Steffensen JF (2016) Sources of variation in oxygen consumption of aquatic animals demonstrated by simulated constant oxygen consumption and respirometers of different sizes. Journal of Fish Biology 88: 51–64. doi: 10.1111/jfb.12851 26768971 10.1111/jfb.12851

[48] Svendsen MBS, Bushnell PG, Steffensen JF (2016) Design and setup of intermittent-flow respirometry system for aquatic organisms. Journal of Fish Biology 88: 26–50. doi: 10.1111/jfb.12797 26603018 10.1111/jfb.12797

[49] Urbina MA, Forster ME, Glover CN (2011) Leap of faith: Voluntary emersion behaviour and physiological adaptations to aerial exposure in a non-aestivating freshwater fish in response to aquatic hypoxia. Physiology and Behavior 103: 240–247. doi: 10.1016/j.physbeh.2011.02.009 21316378 10.1016/j.physbeh.2011.02.009

[50] McKenzie DJ, Serrini G, Piraccini G, Bronzi P, Bolis CL (1996) Effects of diet on responses to exhaustive exercise in Nile tilapia (*Oreochromis nilotica*) acclimated to three different temperatures. Comparative Biochemistry and Physiology A Comparative Physiology 114: 43–50.

[51] Crawley MJ (2012) The R Book: Second Edition: John Wiley and Sons 1–1051 p.

[52] King RB (2002) Predicted and observed maximum prey size—snake size allometry. Functional Ecology 16: 766–772.

[53] Reid NM, Addison SL, Macdonald LJ, Lloyd-Jones G (2011) Biodiversity of active and inactive bacteria in the gut flora of wood-feeding Huhu beetle larvae (*Prionoplus reticularis*). Applied and Environmental Microbiology 77: 7000–7006. doi: 10.1128/AEM.05609-11 21841025 10.1128/AEM.05609-11 PMC3187079

[54] White RSA, Wintle BA, McHugh PA, Booker D, McIntosh AR (2017) Population size-persistence scaling does not asymptote in fish populations experiencing extreme climate variability. Proceedings of the Royal Society of London B Biological Sciences 284. 10.1098/rspb.2017.0826 PMC5474084 28615503

[55] Meredith AS (1985) Metabolism and cutaneous exchange in an amphibious fish Neochanna burrowsius (Phillips). Unpublished PhD thesis, University of Canterbury, Christchurch, New Zealand.

[56] Urbina MA, Glover CN (2012) Should I stay or should I go?: Physiological, metabolic and biochemical consequences of voluntary emersion upon aquatic hypoxia in the scaleless fish *Galaxias maculatus*. Journal of Comparative Physiology B: Biochemical, Systemic, and Environmental Physiology 182: 1057–1067. doi: 10.1007/s00360-012-0678-3 22645056 10.1007/s00360-012-0678-3

[57] Chew SF, Chan NKY, Loong AM, Hiong KC, Tam WL, Ip YK (2004) Nitrogen metabolism in the African lungfish (*Protopterus dolloi*) aestivating in a mucus cocoon on land. Journal of Experimental Biology 207: 777–786. 14747410 10.1242/jeb.00813

[58] S H.W. (1930) Metabolism of the lungfish, *Protopterus aethiopicus*. Journal of Biological Chemistry 88: 97–130.

[59] Winterbourn MJ, Rounick JS, Cowie B (1981) Are New Zealand stream ecosystems really different? New Zealand Journal of Marine and Freshwater Research 15: 321–328.

