# Supplementary material for: Metabolism drives distribution and abundance in extremophile fish
Source: PLoS One. 2017 Nov 27;12(11):e0187597. doi: 10.1371/journal.pone.0187597 (PMC5703508; doi:10.1371/journal.pone.0187597)
Supplement: S1 Fig — (DOC) [file pone.0187597.s001.doc]

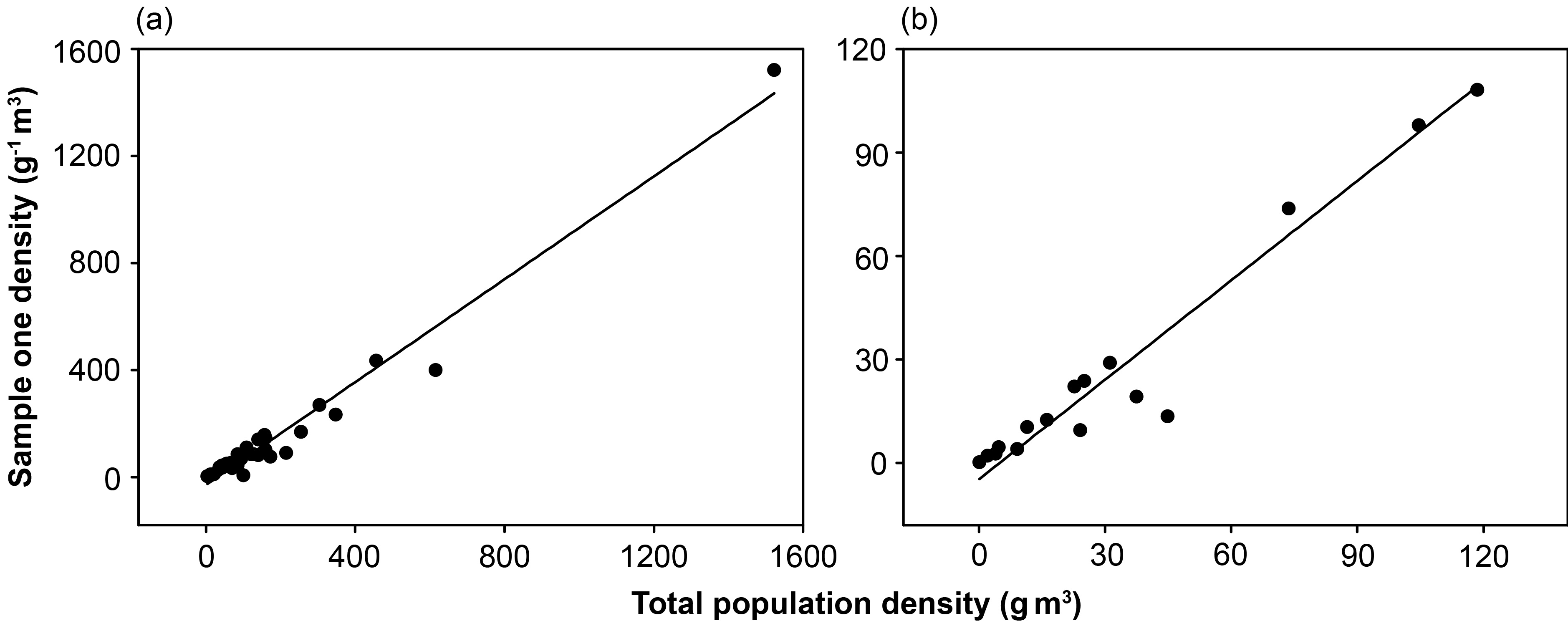


**Fig. S1:** For **(a)** mudfish and **(b)** kokopu, the relationship between the total population density (unique fish biomass from sample one + sample two) and the population density caught on the first sample. Both slopes are 0.95.
